# Impact of COVID-19 pandemic on the Acute Confusional Syndrome by the liaison psychiatry service of Hospital del Mar

**DOI:** 10.1192/j.eurpsy.2022.860

**Published:** 2022-09-01

**Authors:** M. Calls, A. Llimona González, F. Dinamarca, D. García Hernández, S. Oller Canet

**Affiliations:** 1 Hospital del Mar, Psiquiatría, Barcelona, Spain; 2 Parc de Salut Mar, Instituto De Neuropsiquiatría Y Adicciones (inad), Barcelona, Spain

**Keywords:** ACUTE CONFUSIONAL SYNDROME, LIAISON PSYCHIATRY SERVICE, Covid-19

## Abstract

**Introduction:**

The coronavirus disease 2019 (COVID-19) pandemic has had a profound worldwide impact on health. Acute Confusional Syndrome (ACS) is the most common neuropsychiatric complication in COVID-19 infection.

**Objectives:**

Describe the characteristics of the admited patients attended by the liaison psychiatry service for acute confusional syndrome during the COVID 19 pandemic. Sociodemographical and clinical variables were descrived.

**Methods:**

We conducted an observational, descriptive study. All patients attended by the liaison psychiatry service of Hospital del Mar, between February and April 2020, with ACS diagnosis were included.

**Results:**

We included 62 patients with acute confusional syndrom; 35 were men (56.5%), and mean age was 71.71 years (standard deviation [SD]:11.345). The mean duration of admision stay was 41.19 days [SD: 38.039]. The mean number of consultations carried out was 6.5 [SD: 5.422]. 52.5% of our sample of our sample had confusional symptoms for 8 days. 50 patients presented complications during admission (80.6%), of which 43 patients developed infectious complications (69.4%). 59 patients had a history of chronic diseases (95.2%). 54 patiens (88.5%) had potencial risk factors associated with acute confusional syndrome including: isolation in 24 (39.3%), active infection in 46 (74.2%), hypoxemia in 25 (40.3%), previous cognitive impairment in 15 (24.6%)

**Conclusions:**

Acute Confusional Syndrome mainly affects people with risk factors such as isolation, active infection and hypoxemia (which in turn are symptoms of Covid-19).

**Disclosure:**

No significant relationships.

